# Viability of *Trichinella spiralis* in traditional sour pork fermentation and its inactivation by microwave heating: Implications for zoonotic risk and food safety

**DOI:** 10.14202/vetworld.2025.1660-1666

**Published:** 2025-06-19

**Authors:** Atchara Artchayasawat, Benjamabhorn Pumhirunroj, Sukhonthip Khueangchiangkhwang, Thidarut Boonmars, Parichart Boueroy, Porntip Laummaunwai, Panaratana Rattanasuwan

**Affiliations:** 1 Department of Agriculture and Resources, Faculty of Natural Resources and Agro-Industry, Kasetsart University, Chalermphakiat Sakon Nakhon Province Campus, Sakon Nakhon, 47000, Thailand; 2 Cholangiocarcinoma Research Institute, Khon Kaen University, Khon Kaen, 40002, Thailand; 3 Program in Animal Science, Faculty of Agricultural Technology, Sakon Nakhon Rajabhat University, Sakon Nakhon, 47000, Thailand; 4 Department of Parasitology and Infectious Diseases, Gifu University Graduate School of Medicine, Gifu, Japan; 5 Department of Parasitology, Faculty of Medicine, Khon Kaen University, Khon Kaen, 40002, Thailand; 6 Department of Community Health, Faculty of Public Health, Kasetsart University, Chalermphakiat Sakon Nakhon Province Campus, Sakon Nakhon, 47000, Thailand; 7 Department of Anesthesiology, Faculty of Medicine, Khon Kaen University, Khon Kaen, 40002, Thailand

**Keywords:** fermentation safety, foodborne parasitosis, larval viability, microwave heating, sour fermented pork, *Trichinella spiralis*, zoonotic infection

## Abstract

**Background and Aim:**

Cultural dietary practices involving the consumption of raw or undercooked meat, such as traditional sour fermented pork, pose significant risks for foodborne parasitic infections, particularly trichinellosis caused by *Trichinella spiralis*. This study aimed to evaluate the viability of *T. spiralis* larvae during sour pork fermentation and to assess the efficacy of microwave heating as a practical method for inactivating the larvae.

**Materials and Methods:**

Laboratory-bred hamsters were experimentally infected with *T. spiralis* to obtain encysted muscle larvae. Infected muscle samples were incorporated into a traditional sour pork recipe and fermented at ambient temperature (28–30°C) for 5 days. Larval viability was assessed daily using propidium iodide staining and confocal microscopy. In a separate experiment, pork slices embedded with infected muscle were subjected to microwave heating at 400 W (1–4 min) and 800 W (0.5–4.5 min). Post-treatment viability was determined similarly.

**Results:**

Encysted larvae remained viable throughout the 5-day fermentation period, with no uptake of propidium iodide observed in any samples. In contrast, microwave heating at 400 W for 3 min or at 800 W for 1 min or longer resulted in complete larval inactivation, as evidenced by positive staining. Non-heated controls retained viable larvae, while boiling served as an effective positive control for inactivation.

**Conclusion:**

Traditional sour pork fermentation does not inactivate *T. spiralis* larvae within 5 days, underscoring a persistent zoonotic risk. However, microwave heating offers a rapid and accessible intervention for larval inactivation. These findings underscore the significance of public health education and food safety protocols in regions where the consumption of raw meat is culturally prevalent.

## INTRODUCTION

**Figure 1:**
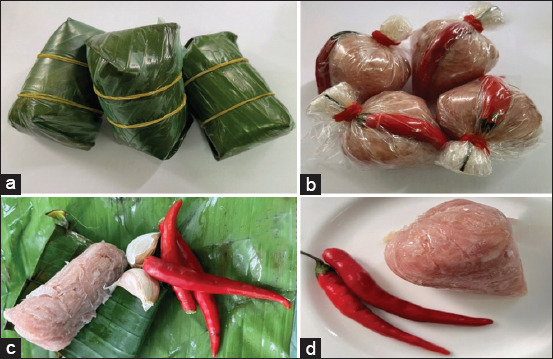
(a–d) A traditional pickled pork or sour fermented pork.

*Trichinella*, the etiological agent of trichinellosis, has been reported in human populations across 55 countries, representing 27.8% of nations globally. Infections with *Trichinella* spp. have also been docu-mented in domestic animals – primarily pigs – in 43 countries (21.9%) and in wildlife in 66 countries (33.3%) [1]. Human trichinellosis primarily arises from the consumption of raw or undercooked meat derived from infected domestic pigs and wild boars containing encysted larvae [2, 3]. Clinical manifestations vary with the stage of infection and typically include diarrhea, facial edema, periorbital hemorrhage, and myalgia [4–6]. The severity of disease is influenced by the infective larval dose and the host’s immune response [7]. Notably, the global incidence of outbreaks has increased in recent years, suggesting a shift in the epidemiological landscape. This rise is largely attributed to the increasing consumption of wild boar meat and the continued adherence to traditional dietary customs that involve raw pork. In Southeast Asia, traditional sour fermented pork dishes remain widely consumed and are readily available in local markets (Figure 1). While such fermented foods offer nutritional and organoleptic value, they may also act as vehicles for foodborne para-sitic infections. In response, international efforts have been initiated to improve the microbiological safety of fermented products. It is commonly assumed that the acidic conditions generated during fermentation may neutralize *Trichinella* larvae, similar to the reduction in viability of *Opisthorchis viverrini* observed in pickled fish preparations [8–10]. Nevertheless, trichinellosis continues to pose a significant public health concern, especially in areas where raw meat consumption rem-ains prevalent.

Despite longstanding awareness of *Trichinella spiralis* as a globally distributed zoonotic parasite, its survival in culturally significant fermented pork products remains inadequately explored. Traditional lactic acid fermentation is widely presumed to reduce the viability of various foodborne parasites due to the lowered pH and microbial competition. However, unlike extensively studied liver fluke species such as *O. viverrini*, there is limited empirical evidence regarding the persistence of *T. spiralis* larvae under real-world fermentation conditions commonly practiced in Southeast Asia. Moreover, most prior investigations have focused on Western meat preservation techniques, such as curing, freezing, and salting, leaving a critical knowledge gap regarding indigenous preparation methods that do not involve thermal treatment. In regions where raw or under-fermented pork is routinely consumed, the assumption that short-term fermentation alone ensures safety may contribute to the underestimation of zoonotic transmission risks. In addition, although thermal inactivation of *T. spiralis* has been well docum-ented using conventional cooking, there is a lack of standardized guidance on the use of microwave heating, a ubiquitous but often inconsistently applied method in domestic settings. These gaps hinder evidence-based public health recommendations and expose populations to preventable parasitic infections.

This study aims to (i) evaluate the viability of *T. spirali*s muscle larvae during traditional sour pork fermentation over a 5-day period and (ii) determine the efficacy of microwave heating at varying power levels and exposure durations as a method for larval inactivation. By simulating real-life culinary practices in Southeast Asia, the study aims to determine whether traditional fermentation can independently neutralize encysted larvae and to what extent microwave treat-ment provides a reliable alternative for enhancing food safety. The findings will contribute to a more accurate assessment of zoonotic risks associated with the consumption of fermented pork and support the development of culturally appropriate, science-based food safety guidelines.

## MATERIALS AND METHODS

### Ethical approval

This study was approved by the Institutional Animal Care and Use Committee of Khon Kaen University, Thailand (ACUC-KKU-51/2561).

### Study period and location

This study was conducted from May 2019 to June 2023 at the Department of Parasitology, Faculty of Medicine, Khon Kaen University, Thailand.

### Animal infection and maintenance

Laboratory-bred hamsters were obtained from the Animal Facility, Faculty of Medicine, Khon Kaen University, Khon Kaen, Thailand. To produce encysted muscle larvae, each hamster was orally administered 200 viable *T. spiralis* larvae suspended in 0.2 mL of physiological saline. The infected animals were main-tained for 45 days post-infection at the animal house of the same faculty [11–13]. Euthanasia was performed through inhalation of 1%–3% isoflurane, adhering to the Institutional Animal Ethics Guidelines.

### Preparation of *T. spiralis*-infected muscle tissue

**Figure 2:**
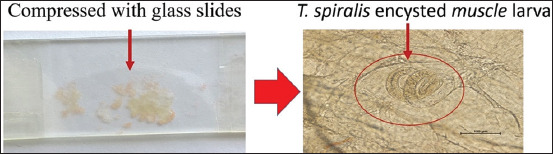
*Trichinella spiralis* encysted muscle larva in minced infected muscle specimens subjected to compression using glass slides and observation under a microscope.

Following euthanasia, skeletal muscles from infected hamsters were excised, finely minced, homogenized, and weighed using a precision digital balance. A 10 mg aliquot of infected muscle was subjected to digestion using artificial gastric juice (1% hydrochloric acid and 1% pepsin) to quantify larval burden. In addition, 100 mg samples of infected tissue were compressed between glass slides and examined microscopically to enumerate encysted larvae (Figure 2). Verified infected muscle samples were allocated to experimental and control groups accordingly.

### Viability controls and propidium iodide staining

**Figure 3:**
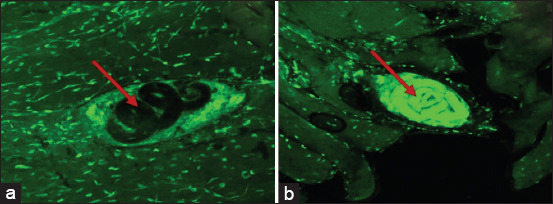
A negative (a - alive larva) and positive (b - dead larva) control of infected muscle post propidium iodide staining. Arrow - larva.

Larval viability was determined using propidium iodide staining as described by Boueroy *et al*. [14]. Infected muscle specimens were incubated with 0.25 mg/mL propidium iodide (Invitrogen, USA) for 30 min, followed by confocal microscopic analysis. Larvae exhibiting uptake of the stain were classified as non-viable (dead), whereas those without staining were considered viable (Figure 3).

### Preparation of traditional fermented pork and larval viability assessment

**Figure 4:**
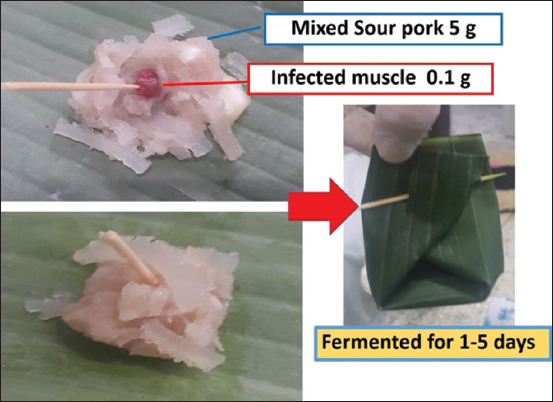
A traditional fermented meat covered with banana leaf.

Traditional sour pork was prepared using a standardized formulation: 400 g minced pork, 300 g pigskin, 25 g salt, 100 g cooked sticky rice, and 120 g garlic. Each 5 g portion of the meat mixture was supplemented with 100 mg of infected muscle, wrapped in banana leaf, and fermented under ambient conditions (temperature 28°C–30°C; relative humidity 60%–80%) for 5 days (Figure 4). Non-fermented infected muscle served as the negative control, and boiled infected muscle was used as the positive control. All groups were evaluated in triplicate. Daily assessment of larval viability was conducted through propidium iodide staining and confocal microscopy. Infected tissues were stained for 30 min with 0.25 mg/mL propidium iodide, and larvae were classified as viable or non-viable based on staining results.

### Microwave heating of infected muscle tissue

**Figure 5:**
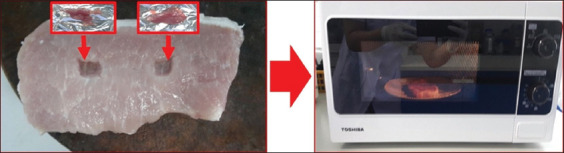
The piece of pork with hole for containing infected muscle (red box) for microwave heating.

To evaluate the impact of microwave heating on larval viability, pork slices were cut to a uniform thickness of 0.5 inches. Each slice was modified to include two central cavities (0.25 inches deep), which were filled with 100 mg of *T. spiralis*-infected minced muscle. The cavities were then sealed with additional pork slices (Figure 5). Samples were microwaved at 400 W for 1, 2, 3, or 4 min, and at 800 W for 30 s, 1 min, or 4.5 min. Following treatment, 100 mg of muscle from each group was stained with 0.25 mg/mL propidium iodide to evaluate larval viability. The untreated infected muscle served as the negative control, while the boiled infected muscle functioned as the positive control. All experimental treatments and controls were performed in triplicate to ensure reproducibility.

## RESULTS

### Viability of *T. spiralis* larvae during traditional fermented pork preparation

**Figure 6:**
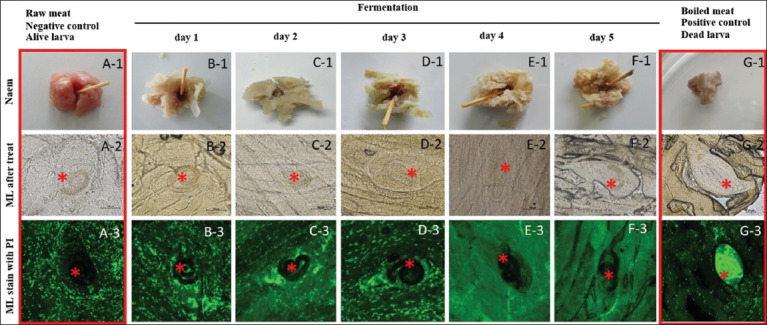
A-1 to G-3: Representative encysted muscle larvae of *Trichinella spiralis* in pickled pork at various time points. The Red Box represents both the negative and positive control groups, while the Red Star indicates the *T*. spiralis larvae.

Microscopic examination revealed that all infected muscle samples contained approximately 6–9 encysted larvae per 100 mg of tissue, as detailed in Table 1. Negative control group consisted of raw infected muscle (Figures 6A-1 and 6A-2). The larvae did not absorb propidium iodide, indicating that they were viable (Figure 6a-3). Conversely, larvae in the positive control group (Figures 6G-1 and 6G-2) displayed clear propidium iodide uptake, confirming non-viability (Figure 6g-3). Throughout the 5-day fermentation period (Figures 6B1–6F3), larvae in all experimental groups failed to absorb the stain, demonstrating persistent viability. These results were consistent with those observed in the negative control group, suggesting that traditional fermentation conditions did not compromise larval survival.

**Table 1:** The survival rate of encysted muscle larvae of *Trichinella spiralis* during the fermentation process.

Fermentation time	Groups	Total number of counts (Mean ± standard deviation)	Percentage of dead larvae	Percentage of survival rate
Day 1	Sour pork	8.33 ± 0.58	0	100
	Raw infected muscle	7.67 ± 0.58	0	100
	Boiled infected muscle	9.33 ± 0.58	100	0
Day 2	Sour pork	8.33 ± 1.53	0	100
	Raw infected muscle	8.33 ± 0.58	0	100
	Boiled infected muscle	6.67 ± 0.58	100	0
Day 3	Sour pork	8 ± 1	0	100
	Raw infected muscle	9.67 ± 0.58	0	100
	Boiled infected muscle	9 ± 0	100	0
Day 4	Sour pork	8 ± 0	0	100
	Raw infected muscle	8.33 ± 0.58	0	100
	Boiled infected muscle	8.67 ± 0.58	100	0
Day 5	Sour pork	8 ± 1	0	100
	Raw infected muscle	9 ± 0	0	100
	Boiled infected muscle	8.67 ± 0.58	100	0

### Larval viability following microwave heating

**Figure 7:**
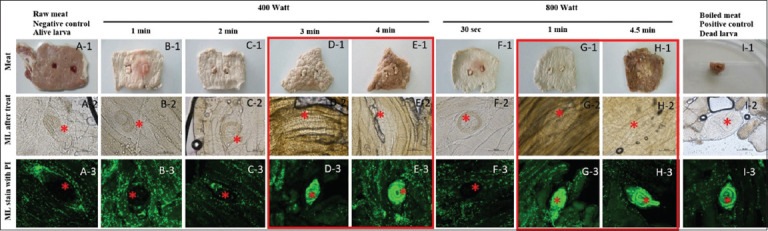
A-1 to I-3: Representative encysted muscle larvae of *Trichinella spiralis* after heating by microwave at various time points. The Red Box represents experimental treatment groups induced with clear propidium iodide, while the Red Star indicates the *T. spiralis* larvae.

As shown in Table 2, the number of encysted larvae remained comparable across all microwave-treated groups. In the raw infected muscle (negative control) (Figures 7A-1 and 7A-2), no propidium iodide staining was observed, indicating larval viability (Figure 7a-3). Similarly, microwave heating at 400 W for 1 min (Figures 7B-1 and 7B-2), and 2 min (Figures 7C-1 and 7C-2), did not result in staining (Figures 7B-3 and 7C-3), indicating that the larvae remained viable under these conditions. However, exposure to 400 W for 3 min (Figures 7D-1, 7D-2 and 7E-1, 7E-2) induced clear propidium iodide staining in the larvae (Figures 7D-3 and 7E-3), signifying effective inactivation. Heating at 800 W for 30 sec did not result in any staining (Figures 7F-1, 7F-2, and 7F-3). Furthermore, extending the heating duration to 1 min (Figures 7G-1 and 7G-2) and 4.5 min (Figures 7H-1 and 7H-2) resulted in complete larval staining (Figures 7G-3 and 7H-3), thereby confirming successful inactivation. In contrast, larvae from the positive control group (boiled muscle) (Figures 7I-1 and 7I-2) also showed complete staining (Figure 7i-3), validating the staining method.

**Table 2:** Effect of microwave heating on *Trichinella spiralis*-infected muscle.

Power of the microwave (Watt)	Time (min)	Total number of counts (Mean ± standard deviation)	Percentage of dead larvae
400	1	8 ± 1.73	0
	2	8 ± 2.65	6.67
	3	10.33 ± 3.51	100
	4	7 ± 1	100
800	0.5	6.67 ± 0.58	0
	1	6.67 ± 2.51	100
	4.5	7.67 ± 1.53	100
Positive control (boiled infected muscle)	10	7.67 ± 2.52	100
Negative control (raw infected muscle)		6.67 ± 1.53	0

## DISCUSSION

### Survivability of *T. spiralis* in fermented pork products

This study is the first to provide empirical evi-dence that traditional lactic fermentation of pork for up to 5 days is insufficient to inactivate encysted *T. spiralis* muscle larvae. This conclusion is supported by the absence of propidium iodide staining, which indicates that larval viability was maintained throughout the fermentation process. In contrast, microwave heating for short durations was found to effectively inactivate the larvae in pork slices of 0.5-inch thickness.

### Public health risks of trichinellosis and cultural food practices

Trichinellosis, also known as trichinosis, is a parasitic infection caused by nematodes of the genus *Trichinella*. Human infection typically results from the consumption of undercooked or raw meat, most commonly pork, that harbors encysted larvae. *T. spiralis* is among the most prevalent species causing human disease through the ingestion of infected meat.

In Southeast Asia, traditional sour fermented pork – a semi-dry, lactic-acid fermented sausage – is a commonly consumed delicacy, particularly in Thailand. This dish is prepared by mixing minced raw pork with sticky rice, garlic, salt, and other seasonings. The lactic fermentation process, often employing <20% salt and using rice as the primary carbohydrate source, is also widely used for fish and other meats in the region. However, this method does not involve heat treatment, which poses a significant risk for foodborne parasitic infections. Consumption of such uncooked fermented pork has been associated with outbreaks of *Taenia* and *Trichinella* infections globally [15, 16]. This risk parallels that posed by pickled fish (Figure 1), a known trans-mission route for *O. viverrini* and a major contributing factor to cholangiocarcinoma in Southeast Asia [17].

### Role of fermentation parameters and parasite inactivation

Despite the longstanding cultural acceptance, the consumption of raw meat remains a health hazard. Public health education is thus crucial to raise awareness of the risks associated with these traditional practices. The antimicrobial effects of fermentation depend heavily on factors such as acidity and salinity. The current findings indicate that fermentation durations of 5 days or less do not affect the viability of *T. spiralis* larvae. In contrast, Hill *et al*. [18] reported complete inactivation of *T. spiralis* under more stringent conditions involving a low pH (<5.2) and extended curing for ≥10 days with 1.3%–2.8% sodium chloride.

Similarly, Oh *et al*. [19] demonstrated the inactivation of *Anisakis* spp. larvae in salt-fermented squid when stored in 15% NaCl for 7 days or 20% NaCl for 6 days. However, substantial viability was still observed in lower salt concentrations: 81.7% in 5% NaCl and 26.7% in 10% NaCl after 7 days of storage. In a related study, Rodriguez-Canul *et al*. [20] showed that *Taenia solium* cysticerci were rendered inactive following salt pickling of infected pork.

### Variability in fermentation efficacy across parasite species

Previous studies have shown that fermentation extending beyond 3 days can successfully eliminate *O. viverrini* and other intestinal flukes from cyprinid fish [9, 10]. However, the effectiveness of lactic fermentation as a decontamination method may vary depending on the parasite species and the structural resilience of their infective stages. Ducrocq *et al*. [21] highlighted that individuals consuming raw or undercooked meat face a 1.2–1.3-fold higher risk – and 1.7–3.0-fold higher odds – of *Toxoplasma gondii* infection compared to those who thoroughly cook meat, regardless of the animal species.

## CONCLUSION

This study provides compelling evidence that traditional lactic fermentation of pork for up to 5 days does not inactivate encysted *T. spiralis* larvae. The viability of larvae throughout the fermentation process was confirmed by the absence of propidium iodide staining. In contrast, microwave heating at 400 W for at least 3 min or at 800 W for 1 min effectively inactivated larvae in pork slices of 0.5-inch thickness, as evidenced by complete larval staining.

These findings have critical practical implications for food safety in regions where traditional raw or under-fermented pork dishes are routinely consumed. Reliance on short-duration fermentation as a standalone method of parasite inactivation is insufficient and may perpetuate the risk of zoonotic transmission. Incorp-orating microwave heating as a rapid and accessible intervention could significantly reduce the risk of infe-ction in domestic settings.

A major strength of this study is its simulation of real-world culinary practices and its use of stand-ardized viability assays under both fermented and thermally treated conditions. The application of confocal microscopy, combined with propidium iodide staining, offered a reliable and sensitive approach for distinguishing between viable and non-viable larvae.

However, the study has certain limitations. First, it focused solely on a 5-day fermentation period and did not assess extended durations or variations in pH and salt concentration that may influence larval viability. Second, the microwave heating experiments were limited to specific power settings and pork thicknesses, which may not encompass all household cooking scenarios. In addition, only *T. spiralis* was evaluated, and the generalizability to other *Trichinella* species or foodborne helminths remains uncertain.

Future research should explore the interaction of fermentation parameters (e.g., pH, NaCl concentration, and fermentation duration) with larval viability and investigate the efficacy of other non-thermal preservation techniques. Expanding inactivation trials to include other *Trichinella* species and various fermented meat matrices will also enhance risk assessment models and inform public health recommendations.

While traditional fermented pork remains a val- ued culinary practice, it poses an underrecognized parasitic risk if not properly handled. This study highlights the urgent need for culturally sensitive food safety education and provides an evidence-based thermal guideline – microwave heating at ≥800 W for 1 min – to ensure the microbiological safety of fermented pork products.

## AUTHORS’ CONTRIBUTIONS

AA, BP, SK, PB, and PL: Data curation, investigation, methodology, and formal analysis. AA and TB: Conceptualization and project administration. TB and PR: Supervision and visualization. AA, TB, and PR: Drafted, reviewed, and revised the manuscript. All authors have read and approved the final manuscript.
